# Brain Metastasis Mimicking Brain Abscess in ALK-Positive Non-Small-Cell Lung Cancer

**DOI:** 10.1155/2019/9141870

**Published:** 2019-06-17

**Authors:** Toshio Sakatani, Hidenori Kage, Shunsaku Takayanagi, Kaoru Watanabe, Yoshihisa Hiraishi, Aya Shinozaki-Ushiku, Shota Tanaka, Tetsuo Ushiku, Nobuhito Saito, Takahide Nagase

**Affiliations:** ^1^Department of Respiratory Medicine, Graduate School of Medicine, University of Tokyo, Tokyo, Japan; ^2^Department of Neurosurgery, Graduate School of Medicine, University of Tokyo, Tokyo, Japan; ^3^Department of Pathology, Graduate School of Medicine, University of Tokyo, Tokyo, Japan

## Abstract

Brain metastasis frequently develops in non-small-cell lung cancer (NSCLC). Here, we report a patient who developed brain metastasis from *ALK*-positive NSCLC which mimicked brain abscess. He was admitted for suspected obstructive pneumonia nine months after curative lung resection. Head magnetic resonance imaging revealed a cavitary lesion, which was compatible with brain abscess but rare in brain metastasis. However, after treatment with antibiotics, the brain lesion increased in size. Aspiration of the liquid content of the brain lesion revealed cancer cells. When a brain lesion suggestive of abscess develops in a patient with *ALK*-positive NSCLC, aspiration may be necessary to differentiate metastasis from abscess.

## 1. Introduction

Non-small-cell lung cancer (NSCLC) is the most common cause of cancer-related mortality. Despite advances in chemotherapy, radiation, and surgery, the prognosis of NSCLC is generally poor, with a 5-year survival rate of 44%. One reason is that NSCLC is a cancer susceptible to brain metastasis. Patients with brain metastases have poor prognosis, with a median overall survival of less than 3 months without treatment [1].

The incidence of brain metastasis in patients with *ALK*-positive NSCLC is high, possibly due to the longer survival achieved with the use of *ALK* inhibitors [2]. A retrospective study has reported that 24% of patients with *ALK*-positive NSCLC had brain metastasis at initial evaluation and 58% at 3 years [3]. Therefore, it is crucial to properly diagnose and treat brain metastasis.

Generally, the diagnosis of brain metastasis is made by imaging studies such as computed tomography (CT) or magnetic resonance imaging (MRI). Diffusion-weighted imaging (DWI) and apparent diffusion coefficient (ADC) can distinguish brain abscess from brain metastasis in suspected cases. Typically, both brain metastasis and brain abscess are T1-weighted image (T1WI) high and T2-weighted image (T2WI) low on MRI. However, brain metastasis is usually DWI low and ADC high, while brain abscess is DWI high and ADC low. Additionally, magnetic resonance spectroscopy (MRS) may assist in making the correct diagnosis when combined with DWI.

Here, we present a patient with *ALK*-positive lung cancer who developed brain metastasis that mimicked brain abscess.

## 2. Case Report

**Figure 1:**
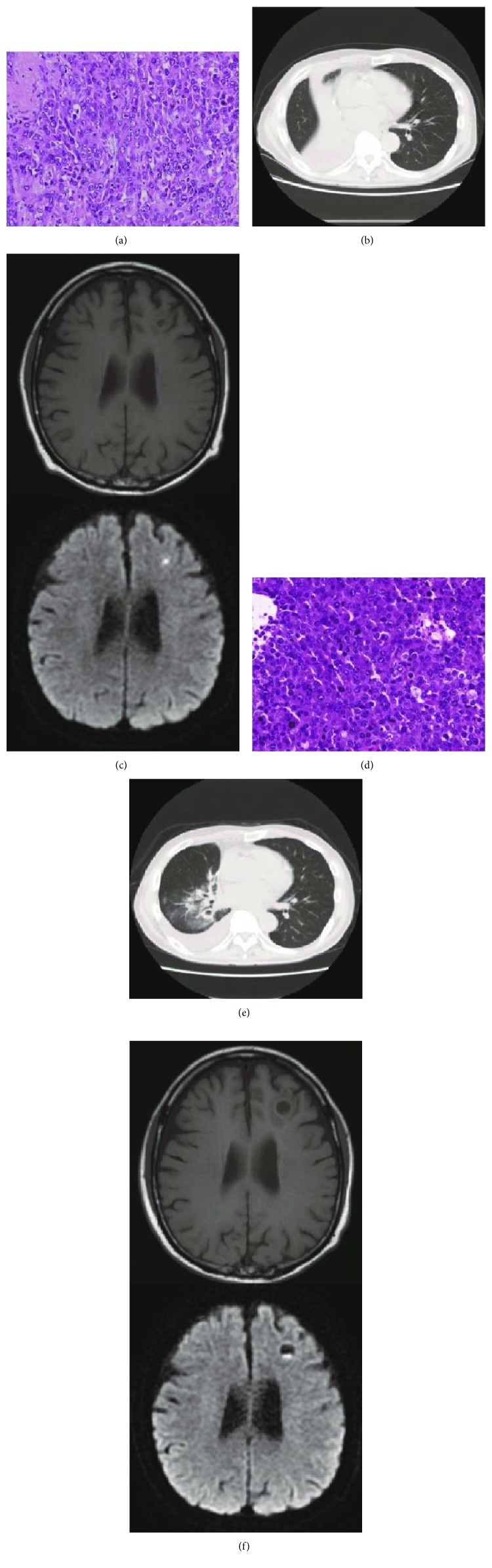
(a) Pathological findings: lung cancer tissue after pulmonary resection (×400 at original magnification). (b) Chest CT taken at admission revealed consolidation and atelectasis in the right lower lobe and right pleural effusion. (c) A 5 mm cranial lesion was found by head MRI at admission (upper panel: T1-weighted MRI, lower panel: diffusion-weighted MRI). (d) Pathological findings: the brain lesion which was resected (×400 at original magnification). (e) Chest CT taken three weeks later showed that consolidation and atelectasis improved, revealing ground glass opacities and multiple cavitary lesions in the right lower lobe. (f) On day 27, the brain lesion increased to 14 mm and exacerbation of cerebral edema around the brain lesion was observed by head MRI (upper panel: T1-weighted MRI, lower panel: diffusion-weighted MRI).

A 68-year-old former smoker was diagnosed with stage IIA (pT2aN0M0) NSCLC after undergoing right middle lobe resection (Figure 1(a)). Immunohistochemistry of lung cancer and fluorescent in situ hybridization revealed *ALK* fusion-positive NSCLC. The bronchial and pulmonary vessel stumps were positive, and additional radiation therapy (56 Gy/7 fractions) was performed. Postoperative adjuvant chemotherapy was not performed because of poor renal function.

Nine months after curative surgery, he was admitted to our hospital due to dyspnea and malaise. White blood cell count (WBC) was 37,000/*μ*l, CRP was 2.6 mg/dl, and procalcitonin was 19.1 ng/ml. Chest CT revealed consolidation and atelectasis in the right lower lobe and right pleural effusion (Figure 1(b)). A 5 mm cranial lesion was also found by head MRI (Figure 1(c)). The cranial lesion had rims that were slightly T1WI high and T2WI low. In addition, the rim was homogeneous and did not infiltrate the surrounding normal brain tissue. Gadolinium enhancement could not be performed due to poor renal function. We suspected obstructive pneumonia and brain abscess and started piperacillin/tazobactam. On day 15, bronchoscopy was performed for possible recurrence of lung cancer, but no cancer cells or bacteria were detected. After 3 weeks of treatment with antibiotics, WBC decreased to 7300/*μ*l and CRP decreased to 3.4 mg/dl after reaching a peak of 10.4 mg/dl. Chest CT revealed that consolidation and atelectasis in the right lower lobe improved, while ground glass opacities and multiple cavitary lesions appeared (Figure 1(e)). On day 27, the brain lesion increased to 14 mm and exacerbation of cerebral edema surrounding the brain lesion was observed, raising the possibility of brain metastasis (Figure 1(f)). On MRI, the content of a cystic lesion partially showed high intensity by DWI and ADC was low, suggesting brain abscess. MRS showed a peak of lactate, but peaks of alanine, succinate, acetate, and pyruvate, characteristic of brain abscess, could not be confirmed. On day 43, the brain lesion increased to 17 mm and cerebral edema worsened. Neurological symptoms were not observed. On day 44, the skull was punctured to aspirate the liquid content of the brain lesion, which revealed cancer cells with many denatured cells as well as inflammatory cells and necrotic substances, while bacterial culture was negative. The patient was diagnosed with recurrence of lung cancer and brain metastasis. On day 49, epilepsy developed and was treated with levetiracetam and phenytoin. On day 51, the brain metastasis was resected (Figure 1(d)), followed by whole brain radiotherapy (30 Gy/10 fractions). Alectinib was started on day 77, and the patient was discharged on day 95.

## 3. Discussion

Brain metastasis is frequently detected in patients with *ALK*-positive NSCLC. Appropriate treatment for brain metastasis is associated with long-term survival in patients with *ALK*-positive NSCLC [4]. With proper diagnosis, local therapy and new drugs with good blood-brain barrier penetration become therapeutic options [5]. Generally, brain metastasis can be differentiated from brain abscess using MRI. However, the brain metastasis in this *ALK*-positive NSCLC patient was similar to brain abscess by brain MRI and MRS.

Brain metastasis from *ALK*-positive NSCLC can present as a cystic lesion [6]. Recently, hemorrhagic brain metastasis was reported in *ALK*-positive NSCLC [7]. Thus, atypical presentation of brain metastasis from *ALK*-positive NSCLC may be common. However, brain metastasis mimicking brain abscess has never been reported.

Clinical presentation can help differentiate brain metastasis from brain abscess. If a patient presents with fever and inflammatory findings, the likelihood of brain abscess increases. However, lung cancer patients commonly have fever and inflammation, because of concomitant infection or from tumor itself. In fact, we initially suspected brain abscess and lung abscess in this patient because he had cavitary lesions in the lung as well as inflammatory findings. Furthermore, there was no evidence of malignancy by bronchoscopy. Neither the clinical course nor imaging studies could rule out brain abscess. MRS did not show peaks of alanine, succinate, acetate, or pyruvate, characteristic of brain abscess, but this could have been due to treatment with antibiotics. Ultimately, aspiration of the liquid content revealed the diagnosis of brain metastasis.

When a brain lesion suggestive of abscess develops in patients with *ALK*-positive NSCLC, aspiration may be necessary to differentiate metastasis from abscess.

## References

[1] Nussbaum E. S., Djalilian H. R., Cho K. H., Hall W. A. (1996). Brain metastases: histology, multiplicity, surgery, and survival. *Cancer*.

[2] Gadgeel S. M., Gandhi L., Riely G. J. (2014). Safety and activity of alectinib against systemic disease and brain metastases in patients with crizotinib-resistant ALK-rearranged non-small-cell lung cancer (AF-002JG): results from the dose-finding portion of a phase 1/2 study. *The Lancet Oncology*.

[3] Rangachari D., Yamaguchi N., VanderLaan P. A. (2015). Brain metastases in patients with EGFR-mutated or ALK-rearranged non-small-cell lung cancers. *Lung Cancer*.

[4] Johung K. L., Yeh N., Desai N. B. (2016). Extended survival and prognostic factors for patients with ALK-rearranged non-small-cell lung cancer and brain metastasis. *Journal of Clinical Oncology*.

[5] Zhang I., Zaorsky N. G., Palmer J. D., Mehra R., Lu B. (2015). Targeting brain metastases in ALK-rearranged non-small-cell lung cancer. *The Lancet Oncology*.

[6] Kim S.-H., Hyun J.-W., Kim H. J. (2017). De novo cystic brain lesions mimicking neurocysticercosis in ALK- positive lung cancer. *Lung Cancer*.

[7] Shi M., Xu H., DiPoto Brahmbhatt A., Gonzalez-Toledo E., Georgescu M. M. (2018). Hemorrhagic brain metastases in a patient with anaplastic lymphoma kinase (ALK)-rearranged invasive mucinous adenocarcinoma of the lung. *American Journal of Case Reports*.

